# Molecular characteristics of endometrial cancer coexisting with peritoneal malignant mesothelioma in Li-Fraumeni-like syndrome

**DOI:** 10.1186/s12885-015-1010-x

**Published:** 2015-01-15

**Authors:** Angel Chao, Chyong-Huey Lai, Yun-Shien Lee, Shir-Hwa Ueng, Chiao-Yun Lin, Tzu-Hao Wang

**Affiliations:** 1 Department of Obstetrics and Gynecology, Chang Gung Memorial Hospital and Chang Gung University, Taoyuan, Taiwan; 2 Genomic Medicine Research Core Laboratory, Chang Gung Memorial Hospital, Taoyuan, Taiwan; 3 Department of Biotechnology, Ming-Chuan University, Taoyuan, Taiwan; 4 Department of Pathology, Chang Gung Memorial Hospital and Chang Gung University, Taoyuan, Taiwan

**Keywords:** Endometrial cancer, Peritoneal malignant mesothelioma, Massively parallel DNA sequencing, Molecular inversion probe microarray

## Abstract

**Background:**

Endometrial cancer that occurs concurrently with peritoneal malignant mesothelioma (PMM) is difficult to diagnose preoperatively.

**Case presentation:**

A postmenopausal woman had endometrial cancer extending to the cervix, vagina and pelvic lymph nodes, and PMM in bilateral ovaries, cul-de-sac, and multiple peritoneal sites. Adjuvant therapies included chemotherapy and radiotherapy. Targeted, massively parallel DNA sequencing and molecular inversion probe microarray analysis revealed a germline TP53 mutation compatible with Li-Fraumeni-like syndrome, somatic mutations of PIK3CA in the endometrial cancer, and a somatic mutation of GNA11 and JAK3 in the PMM. Large-scale genomic amplifications and some deletions were found in the endometrial cancer. The patient has been stable for 24 months after therapy. One of her four children was also found to carry the germline TP53 mutation.

**Conclusions:**

Molecular characterization of the coexistent tumors not only helps us make the definite diagnosis, but also provides information to select targeted therapies if needed in the future. Identification of germline TP53 mutation further urged us to monitor future development of malignancies in this patient and encourage cancer screening in her family.

**Electronic supplementary material:**

The online version of this article (doi:10.1186/s12885-015-1010-x) contains supplementary material, which is available to authorized users.

## Background

Simultaneous occurrence of primary cancers from the breast, ovary, and colon has been reported with endometrial cancer [1]. However, coexistence of mesothelioma with endometrial cancer is rare with only three reported cases [2–4]. The diagnosis of endometrial cancer is usually made via endometrial curettage prior to definitive surgery whereas the mesothelioma would not be discovered until surgery.

In the present study, we describe a patient with advanced endometrial cancer and a concomitant peritoneal malignant mesothelioma (PMM). Hematoxylin and eosin stains and the immunoprofile of tumor tissues differentiated the two tumors. Furthermore, we used targeted, massively parallel sequencing (MPS) and molecular inversion probe (MIP)-based microarray analysis to molecularly characterize these tumors. The genetic make-up of the concomitant tumors of this patient was determined, and identification of a germline TP53 mutation indicating Li-Fraumeni-like syndrome in this patient encouraged further screening for this mutation in her family members.

## Case presentation

**Figure 1:**
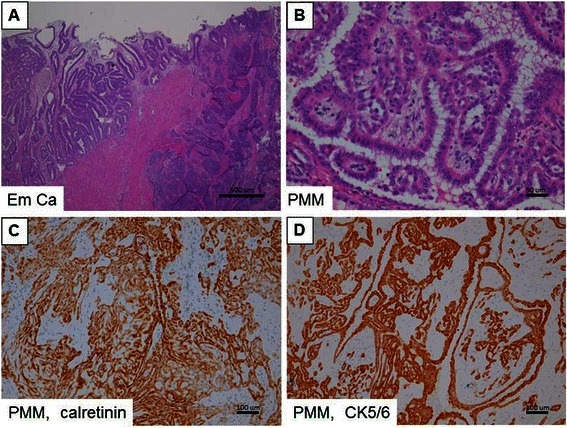
**Hematoxylin and eosin (H&E) stains and immunohistochemical studies for two primary cancers. (A)** H&E stain; endometrial cancer. **(B)** H&E stain; peritoneal malignant mesothelioma (PMM). High expression of **(C)** calretinin and **(D)** CK5/6 is shown in brown color in PMM. Scale bars represent 500 μm in **(A)**, 50 μm in **(B)**, and 100 μm in **(C & D)**.

A 54-year-old female patient, gravida 5, para 4, with the history of one elective abortion, suffered from abnormal postmenopausal bleeding for two weeks. She was referred from a local clinic where the diagnosis of adenocarcinoma was made based on the results of a cervical biopsy. She had no known asbestos exposure. Her father died of hepatocellular carcinoma at 70 years of age. Pelvic examination showed a necrotic cervical tumor that extended posteriorly, shortening the posterior fornix. Pathology indicated that the cervical tumor was a poorly-differentiated adenocarcinoma, which was carcinoembryonic antigen (CEA) (-), vimentin (+), estrogen receptor (ER) (1+), and progesterone receptor (PR) (3+). The results were compatible with an endometrial origin. Magnetic resonance imaging (MRI) revealed a mass invading the endometrium and both cervical lips as well as tumor implants in the pouch of Douglas and the supravesical pouch. Positron emission tomography (PET) showed fluorodeoxyglucose uptake in the cervix and uterus. Her surgical staging, using the International Federation of Gynecology and obstetrics (FIGO) guidelines, was IIb. Final pathologic diagnosis was endometrioid adenocarcinoma with cervical extension, positive vaginal margin, and bilateral pelvic lymph node metastases, upstaging the malignancy to FIGO stage IIIc, grade 3. Intriguingly, a co-existent mesothelioma, T2N0M0, was discovered extended to bilateral ovaries, peritoneum, omentum, multiple mesenteric sites, cul-de-sac, and bladder serosa. Residual disease of mesothelioma of less than 5 mm was noted. These mesothelioma tissues were positive for calretinin and cytokeratin (CK)5/6, but negative for ER, PR, and epithelial cell adhesion molecule (EpCAM, Ber-EP4) (Figure 1). CK5/6 and calretinin are often positive in mesothelial cells and mesothelial tumors, but negative in adenocarcinoma. Ber-EP4, on the other hand, is frequently positive in adenocarcinoma, but negative in mesothelial cells. Therefore, the immunostaining results supported the diagnosis of mesothelioma.

The patient received chemotherapy with cisplatin (75 mg/m^2^) and premetrexed (Alimta, 500 mg/m^2^) every 3 weeks for a total of 6 cycles. She also received concurrent radiation to the pelvis (5040 cGy) with brachytherapy (200 cGy) for cervical extension and positive vaginal margins of the endometrial cancer. Subsequently, small field radiation therapy was given to the hepato-renal region (6000 cGy/25 fractions) to treat the PMM. The patient has remained clinically stable for 24 months though her follow-up computed tomography did show stationary lesions at Morrison’s pouch at the 24-month follow-up visit.

For molecular diagnosis, formalin-fixed paraffin-embedded (FFPE) tissues of these two tumors and blood DNA of the patient were sequenced using targeted MPS (TruSeq Amplicon Cancer Panel, Illumina). Genomic profiling of tumor tissues was done with MIP-based microarray analysis (Oncoscan, Affymetrix). The results are summarized in Table 1. Detailed results from the MPS are listed in Additional file 1, while the MIP-based microarray analyses are in Additional files 2 and 3. The TP53 mutation (Pro72Arg) found in blood and both tumors was confirmed with Sanger sequencing. Of note, among 4 somatic point mutations in this case, only the PIK3CA mutation (c.1624 G > A p.E542K) was detected by both MPS and MIP microarray.

**Table 1:** **Summary of mutated genes in endometrial cancer (Em Ca) and peritoneal malignant mesothelioma (PMM) measured using massively parallel sequencing (MiSeq) and molecular inversion probe microarray (OncoScan)**

				Em Ca (35%)^‡^		PMM (70%)^‡^	
Gene	Code	Amino acid change	Chromosome	MiSeq sequencing^#^	OncoScan MIP microarray^ξ^	MiSeq sequencing^#^	OncoScan MIP microarray^ξ^
TP53*	215G > C	Pro/Arg	17	+ (55%)	NA	+ (33%)	NA
PIK3CA	1624G > A	Glu/Lys	3	+ (34%)	+	-	-
GNA11	973 T > C	Tyr/His	19	-	NA	+ (32%)	NA
JAK3	703C > T	Ser/Asn	19	-	NA	+ (44%)	NA

*Abbreviation:MIP* molecular inversion probe.

*This mutation is also found in patient’s blood.

^‡^Numbers in the brackets indicate the percentage of tumor cells observed by the pathologist.

^#^Numbers indicate the percentage of alternate alleles.

^ξ^Analyzed by “somatic mutation viewer” (Additional file 3), NA: data not available.

**Figure 2:**
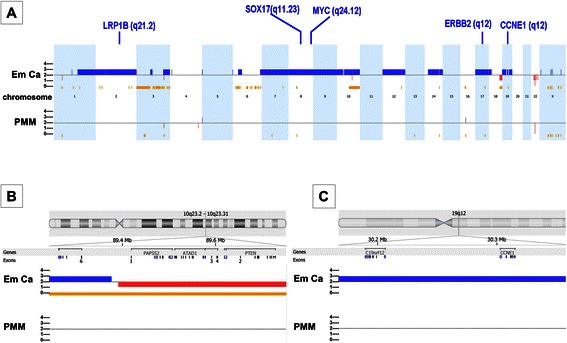
**Schematic representation of the genomic alterations in endometrial cancer (Em Ca, the upper panel) and peritoneal malignant mesothelioma (PMM, the lower panel) detected with molecular inversion probe microarrays. (A)** Copy number gains are shown in blue and losses in red. The genomic regions with loss of heterozygosity are marked in yellow color. Important genes with high frequency of amplification reported in The Cancer Genome Atlas Research dataset of endometrial cancer are also present in this case. **(B)** Detailed genomic alterations are shown in chromosome 10q23-2-23.31, where a tumor suppressor gene PTEN was deleted in endometrial cancer. **(C)** Detailed genomic alterations are shown in chromosome 19q12, where cyclin E1 gene (CCNE1) was amplified in endometrial cancer.

Large regions of amplification in chromosomes 1q21.1-1qter, 2, 7, 8, 9, 10, 12, 14q11.2-14pter, 17, 19p13.3-19p12, and 19p13.2-19q13.42, deletions in 18q22.1-18q23, and loss-of-heterozygosity (LOH) in 3p26.3-3p14.1 and 10q22.1-10q26.3 were detected in the endometrial cancer using the Nexus 6 Copy Number software algorithms for the MIP microarray (Figure 2 and Additional file 2). Only a small number of copy number aberrations and LOH were present in the mesothelioma tumor cells (amplification in 16p11.2 and LOH in 3p21.31-3p21.1). Frequent genomic amplifications reported by The Cancer Genome Atlas Research Network (TCGA) [5], including LRP1B (2q21.2), MYC (8q24.12), SOX17 (8q11.23), ERBB2 (17q12), and CCNE1 (19q12), were also found in the endometrial cancer cells.

**Figure 3:**
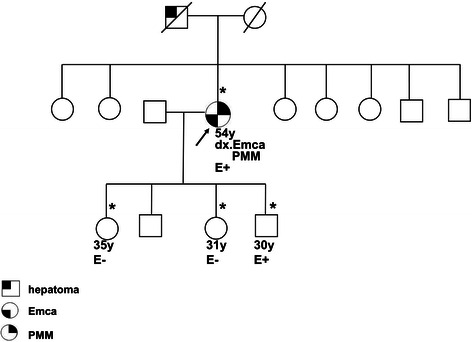
**Gene testing showed the proband (arrow) and one son were heterozygous for the TP53 germline mutation [E+ (mut/wt)].** Her two daughters were homozygous for the wild-type TP53 gene (E-). ‘E’ represents test information on the pedigree. The arrow indicates the proband. The asterisk indicates the kindred who had been examined for the TP53 gene mutation. Abbreviations: dx., diagnosis as; Emca, endometrial cancer; PMM, peritoneal malignant mesothelioma; y, years old. The pedigree was generated according to the recommendations for Pedigree Standardization Task Force of the National Society of Genetic Counselors.

The germline TP53 mutation (Pro72Arg) was compatible with Li-Fraumeni-like syndrome. After genetic counseling with her family members, three of her four children agreed to genetic testing for this mutation. One of her sons was found to carry the TP53 germline mutation [E+ (mut/wt)], but her two daughters were negative for the mutation (E-) (Figure 3) [6].

### Discussion

To our knowledge, this is the first case of endometrial cancer complicated by PMM where molecular characterization has been performed. Immunohistochemical results of calretinin (+), CK5/6 (+), ER (-), and PR (-) in PMM were instrumental in differentiating between the two tumors. A negative Ber-EP4 suggests that the PMM was not a tumor metastasized from the gastrointestinal tract [7].

Surgery for advanced endometrial cancer is challenging. Optimal cytoreduction provides a survival benefit for patients with advanced endometrial cancer [1]. However, the accurate preoperative evaluation of the disease extent to predict the demands of optimal debulking during primary surgery remains difficult. In this case, preoperative imaging studies of MRI and PET did not show evidence of lymph node metastasis. Concurrence of another second primary malignancy further complicated treatment course. In this case, extensive peritoneal implants were mistakenly considered as tumor metastasis. Therefore, the findings of peritoneal metastatic sites during operation should remind operators of the possibility of a co-existing primary cancer. Adjuvant therapies of patients with concurrent endometrial cancer and PMM have not yet been standardized. Radiation therapy has been reported to be the adjuvant therapy of choice, and effective systemic chemotherapies have improved response rates in endometrial cancer [1,8]. Premetrexed has been used in combination with a platinum-base chemotherapy as the treatment for PMM [7].

Because the diagnosis of multiple concurrent primary cancers is often made based on micropathological results, the tissues available for further molecular characterization are in FFPE blocks. For FFPE DNA, targeted MPS [9] and MIP microarrays [10] have proved to be useful in analyzing genomic alterations. Targeted MPS can detect mutations in 48 cancer related genes (Additional file 1). Copy number variations (gains or losses) and LOH in cancer genomes can be further identified using MIP microarrays. Her endometrial cancer had somatic cancer mutations in PIK3CA, which is frequently reported in endometrial cancer [5]. Identification of these mutations may have therapeutic implications. For instance, dysfunctions in the PIK3CA pathways have been successfully treated with PI3K/AKT/mTOR pathway inhibitors [11]. Though copy number variations were found only in small genomic regions of PMM, it harbored GNA11. Mutation of GNA11 in uveal melanoma [12] leads to the activation of downstream mitogen-activated protein kinase (MAPK/ERK) pathways. Clinical trials of inhibitors that disrupt MAPK pathways are being assessed in the treatment of metastatic uveal melanoma [12]. Given the fast pace of current development of targeted drugs aiming at common cancer mutated genes, the identification of multiple mutations in this case may justify her enrollment in future clinical trials of targeted therapy.

Germline TP53 mutations have been reported in 80% of families who have classical Li-Fraumeni syndrome, and 20-40% of families who meet the criteria for Li- Fraumeni-like syndrome [13]. The prevalence of Li-Fraumeni syndrome is rare, with approximately 400 families reported worldwide [14]. However, Li-Fraumeni syndrome may be under reported due to the strict requirement of clinical features and detailed family histories to make the diagnosis.

The Pro72Arg variants of TP53 has been associated with various cancer susceptibility and poor outcomes [15], such as human papillomavirus 16-positive cervical cancer [16], laryngeal tumors [17], head and neck cancers [18], non-small cell lung cancer [19], and bladder cancer [20]. Hisada et al reported an increased risk of developing second cancer in patients with a germline TP53 mutation, with a cumulative probability of 57% of developing a second cancer at 30 years after diagnosis of the first cancer [21]. Clinical management of the patient may include tumor surveillance of other organs such as breast examination [22]. It is advisable that family members take a systematic approach to cancer screening and participate in TP53 research programs [22].

## Conclusions

In conclusion, concurrent endometrial cancer and PMM are difficult to be diagnosed pre-operatively. Effective treatments for such patients are not standardized, and the prognosis is unclear. Molecular characterization of the coexistent tumors not only helps us make definitive diagnosis but also provides information for choosing targeted therapies in the future. In cases of Li-Fraumeni-like syndrome, family members should also be enrolled in cancer screening.

## Consent

Written informed consents were obtained from the patient and her kindred, to publish this case report and accompanying images.

## Additional files

Additional file 1:
**Detailed information of TruSeq Amplicon Cancer Panel.**

Additional file 2:
**The copy number variation (CNV) and loss of heterozygosity (LOH) events in the endometrial cancer (Em Ca) and peritoneal malignant mesothelioma (PMM) identified with the Oncoscan platform.**

Additional file 3:
**Interpretation of somatic mutation with Affymetrix OncoScan microarrays.**
