# Supplementary material for: Molecular characteristics of endometrial cancer coexisting with peritoneal malignant mesothelioma in Li-Fraumeni-like syndrome
Source: BMC Cancer. 2015 Jan 15;15:8. doi: 10.1186/s12885-015-1010-x (PMC4312462; doi:10.1186/s12885-015-1010-x)
Supplement: Additional file 2: — The copy number variation (CNV) and loss of heterozygosity (LOH) events in the endometrial cancer (Em Ca) and peritoneal malignant mesothelioma (PMM) identified with the Oncoscan platform. [file 12885_2015_1010_MOESM2_ESM.doc]

**Additional file 2. The copy number variation (CNV) and loss of heterozygosity (LOH) events in the endometrial cancer (Em Ca) and peritoneal malignant mesothelioma (PMM) identified with the Oncoscan platform.** The MIP microarray, which contains 217,611 probe sets, has been used for identification of CNV (including CN gain and CN loss) and LOH in 891 cancer-related genes, and analysis of 76 selected somatic mutations . CNV calling is based on array-based method. The intensity of each probe is compared with the control (CN = 2). If a tumor probe has CN = 3, the log_2_ ratio is log_2_(3/2). If a tumor probe has CN = 1, the log_2_ ratio is log_2_(1/2). Probe Median indicates the median in the log2 ratio value of the probes in CNV region.

| **Sample** | **Chromosome Region** | **Event** | **Length** | **Cytoband** | **Probe Median** | **Probes number** |
| --- | --- | --- | --- | --- | --- | --- |
| Em Ca | chr1:49,113,622-53,280,177 | LOH | 4166556 | p33 - p32.3 | -0.10 | 323 |
| Em Ca | chr1:52,204,780-53,051,020 | CN Loss | 846241 | p32.3 | -0.22 | 60 |
| Em Ca | chr1:110,231,909-110,269,001 | CN Gain | 37093 | p13.3 | 0.42 | 18 |
| Em Ca | chr1:117,516,341-121,350,934 | LOH | 3834594 | p13.1 - p11.2 | -0.06 | 304 |
| Em Ca | chr1:144,009,053-144,935,753 | CN Gain | 926701 | q21.1 | 0.31 | 18 |
| Em Ca | chr1:154,156,823-157,150,904 | LOH | 2994082 | q21.3 - q23.1 | 0.43 | 343 |
| Em Ca | chr1:144,936,742-241,946,294 | CN Gain | 97009553 | q21.1 - q43 | 0.44 | 7430 |
| Em Ca | chr1:241,951,703-249,212,878 | CN Gain | 7261176 | q43 - q44 | 0.38 | 639 |
| Em Ca | chr2:25,832,117-28,603,637 | LOH | 2771521 | p23.3 - p23.2 | 0.17 | 184 |
| Em Ca | chr2:21,494-61,116,360 | CN Gain | 61094867 | p25.3 - p16.1 | 0.18 | 4771 |
| Em Ca | chr2:36,985,579-40,062,617 | LOH | 3077039 | p22.2 - p22.1 | 0.18 | 292 |
| Em Ca | chr2:62,219,538-141,242,511 | CN Gain | 79022974 | p15 - q22.1 | 0.17 | 5248 |
| Em Ca | chr2:141,261,724-141,496,284 | CN Gain | 234561 | q22.1 | 0.22 | 114 |
| Em Ca | chr2:141,535,561-141,958,235 | CN Gain | 422675 | q22.1 | 0.14 | 271 |
| Em Ca | chr2:141,968,126-142,075,788 | CN Gain | 107663 | q22.1 | 0.27 | 50 |
| Em Ca | chr2:142,087,953-210,314,046 | CN Gain | 68226094 | q22.1 - q34 | 0.18 | 4885 |
| Em Ca | chr2:197,331,508-199,983,384 | LOH | 2651877 | q32.3 - q33.1 | 0.13 | 181 |
| Em Ca | chr2:210,331,533-212,496,352 | CN Gain | 2164820 | q34 | 0.28 | 231 |
| Em Ca | chr2:212,796,443-215,706,075 | LOH | 2909633 | q34 - q35 | 0.20 | 256 |
| Em Ca | chr2:212,499,275-243,052,331 | CN Gain | 30553057 | q34 - q37.3 | 0.19 | 2345 |
| Em Ca | chr3:63,411-63,967,900 | LOH | 63904490 | p26.3 - p14.1 | 0.02 | 5109 |
| Em Ca | chr3:64,033,043-74,819,692 | LOH | 10786650 | p14.1 - p12.3 | 0.07 | 1091 |
| Em Ca | chr3:75,960,298-81,617,839 | LOH | 5657542 | p12.3 - p12.2 | 0.07 | 520 |
| Em Ca | chr3:82,083,260-94,345,533 | CN Gain | 12262274 | p12.2 - q11.2 | 0.10 | 652 |
| Em Ca | chr3:96,728,412-102,528,107 | LOH | 5799696 | q11.2 - q12.3 | 0.09 | 401 |
| Em Ca | chr3:105,374,702-110,517,864 | LOH | 5143163 | q13.11 - q13.13 | 0.04 | 402 |
| Em Ca | chr3:113,711,153-123,188,936 | LOH | 9477784 | q13.31 - q21.1 | 0.06 | 729 |
| Em Ca | chr3:123,804,254-143,439,739 | LOH | 19635486 | q21.2 - q24 | 0.06 | 1482 |
| Em Ca | chr3:147,918,738-150,677,914 | LOH | 2759177 | q24 - q25.1 | 0.07 | 196 |
| Em Ca | chr3:152,575,924-158,864,418 | LOH | 6288495 | q25.2 - q25.32 | 0.10 | 458 |
| Em Ca | chr3:158,296,916-162,440,045 | CN Gain | 4143130 | q25.32 - q26.1 | 0.27 | 289 |
| Em Ca | chr3:162,460,238-162,719,684 | CN Loss | 259447 | q26.1 | -0.54 | 18 |
| Em Ca | chr3:161,477,076-165,952,822 | LOH | 4475747 | q26.1 | 0.27 | 299 |
| Em Ca | chr3:162,726,432-189,388,166 | CN Gain | 26661735 | q26.1 - q28 | 0.26 | 2058 |
| Em Ca | chr3:180,449,758-185,277,794 | LOH | 4828037 | q26.33 - q27.2 | 0.20 | 333 |
| Em Ca | chr3:189,391,544-192,531,202 | LOH | 3139659 | q28 - q29 | 0.22 | 308 |
| Em Ca | chr3:189,391,544-197,852,564 | CN Gain | 8461021 | q28 - q29 | 0.23 | 708 |
| Em Ca | chr3:194,783,938-197,852,564 | LOH | 3068627 | q29 | 0.15 | 253 |
| Em Ca | chr4:11,445,186-11,700,993 | CN Gain | 255808 | p15.33 | 0.23 | 22 |
| Em Ca | chr5:1,296,486-1,364,439 | CN Gain | 67954 | p15.33 | 0.59 | 28 |
| Em Ca | chr5:1,366,748-1,722,978 | CN Gain | 356231 | p15.33 | 0.48 | 31 |
| Em Ca | chr6:204,909-668,627 | CN Gain | 463719 | p25.3 | 0.15 | 53 |
| Em Ca | chr6:16,677,571-20,706,230 | LOH | 4028660 | p22.3 | 0.05 | 359 |
| Em Ca | chr6:23,576,589-27,278,020 | LOH | 3701432 | p22.3 - p22.1 | 0.06 | 253 |
| Em Ca | chr6:28,309,569-40,260,904 | LOH | 11951336 | p22.1 - p21.2 | 0.07 | 1097 |
| Em Ca | chr6:32,288,190-54,727,075 | CN Gain | 22438886 | p21.32 - p12.1 | 0.11 | 1729 |
| Em Ca | chr6:44,473,875-47,343,809 | LOH | 2869935 | p21.1 - p12.3 | 0.07 | 192 |
| Em Ca | chr6:81,030,744-85,794,985 | LOH | 4764242 | q14.1 - q14.3 | 0.06 | 341 |
| Em Ca | chr6:85,966,113-91,153,009 | LOH | 5186897 | q14.3 - q15 | 0.04 | 390 |
| Em Ca | chr6:107,012,077-114,225,811 | LOH | 7213735 | q21 | 0.05 | 647 |
| Em Ca | chr6:130,197,458-140,196,406 | LOH | 9998949 | q22.33 - q24.1 | 0.05 | 741 |
| Em Ca | chr6:146,759,364-151,062,360 | LOH | 4302997 | q24.3 - q25.1 | 0.06 | 295 |
| Em Ca | chr6:153,478,891-156,722,460 | LOH | 3243570 | q25.2 - q25.3 | 0.06 | 221 |
| Em Ca | chr6:163,994,956-167,398,952 | LOH | 3403997 | q26 - q27 | 0.03 | 234 |
| Em Ca | chr6:163,150,310-170,913,051 | CN Gain | 7762742 | q26 - q27 | 0.11 | 550 |
| Em Ca | chr7:41,421-8,288,814 | CN Gain | 8247394 | p22.3 - p21.3 | 0.29 | 691 |
| Em Ca | chr7:8,302,284-11,419,104 | CN Gain | 3116821 | p21.3 | 0.40 | 216 |
| Em Ca | chr7:11,444,666-18,132,062 | CN Gain | 6687397 | p21.3 - p21.1 | 0.38 | 474 |
| Em Ca | chr7:18,148,091-18,563,178 | CN Gain | 415088 | p21.1 | 0.54 | 28 |
| Em Ca | chr7:18,568,627-55,099,836 | CN Gain | 36531210 | p21.1 - p11.2 | 0.34 | 2744 |
| Em Ca | chr7:55,100,083-55,120,926 | CN Gain | 20844 | p11.2 | 0.41 | 34 |
| Em Ca | chr7:55,121,108-56,514,453 | CN Gain | 1393346 | p11.2 | 0.40 | 230 |
| Em Ca | chr7:56,878,859-63,297,158 | CN Gain | 6418300 | p11.2 - q11.21 | 0.14 | 113 |
| Em Ca | chr7:63,311,682-64,231,596 | CN Gain | 919915 | q11.21 | 0.32 | 55 |
| Em Ca | chr7:64,235,566-75,181,645 | CN Gain | 10946080 | q11.21 - q11.23 | 0.21 | 718 |
| Em Ca | chr7:75,182,802-82,823,924 | CN Gain | 7641123 | q11.23 - q21.11 | 0.34 | 566 |
| Em Ca | chr7:82,838,670-83,749,943 | CN Gain | 911274 | q21.11 | 0.42 | 66 |
| Em Ca | chr7:83,774,499-87,418,861 | CN Gain | 3644363 | q21.11 - q21.12 | 0.32 | 303 |
| Em Ca | chr7:87,426,853-88,022,573 | CN Gain | 595721 | q21.12 | 0.24 | 42 |
| Em Ca | chr7:88,026,221-99,489,571 | CN Gain | 11463351 | q21.12 - q22.1 | 0.35 | 879 |
| Em Ca | chr7:99,505,223-102,462,636 | CN Gain | 2957414 | q22.1 | 0.22 | 237 |
| Em Ca | chr7:102,471,345-103,959,006 | CN Gain | 1487662 | q22.1 - q22.2 | 0.29 | 97 |
| Em Ca | chr7:125,790,458-128,323,034 | LOH | 2532577 | q31.33 - q32.1 | 0.32 | 301 |
| Em Ca | chr7:104,334,647-155,530,363 | CN Gain | 51195717 | q22.2 - q36.3 | 0.32 | 4182 |
| Em Ca | chr7:155,532,361-156,772,057 | CN Gain | 1239697 | q36.3 | 0.24 | 83 |
| Em Ca | chr7:156,779,166-159,118,443 | CN Gain | 2339278 | q36.3 | 0.38 | 173 |
| Em Ca | chr8:172,417-49,863,619 | CN Gain | 49691203 | p23.3 - q11.21 | 0.34 | 4139 |
| Em Ca | chr8:46,896,972-51,419,903 | LOH | 4522932 | q11.1 - q11.21 | 0.33 | 370 |
| Em Ca | chr8:49,884,817-50,431,035 | CN Gain | 546219 | q11.21 | 0.24 | 38 |
| Em Ca | chr8:50,445,456-128,743,929 | CN Gain | 78298474 | q11.21 - q24.21 | 0.37 | 6421 |
| Em Ca | chr8:128,745,100-146,292,734 | CN Gain | 17547635 | q24.21 - q24.3 | 0.58 | 1360 |
| Em Ca | chr9:204,738-5,010,091 | CN Gain | 4805354 | p24.3 - p24.1 | 0.24 | 332 |
| Em Ca | chr9:5,071,049-8,519,250 | CN Gain | 3448202 | p24.1 | 0.20 | 434 |
| Em Ca | chr9:8,557,264-21,865,842 | CN Gain | 13308579 | p24.1 - p21.3 | 0.21 | 1062 |
| Em Ca | chr9:22,023,725-33,521,992 | CN Gain | 11498268 | p21.3 - p13.3 | 0.21 | 845 |
| Em Ca | chr9:33,862,508-34,336,905 | CN Gain | 474398 | p13.3 | 0.29 | 32 |
| Em Ca | chr9:34,364,299-76,341,654 | CN Gain | 41977356 | p13.3 - q21.13 | 0.17 | 710 |
| Em Ca | chr9:76,355,347-76,593,162 | CN Gain | 237816 | q21.13 | 0.26 | 18 |
| Em Ca | chr9:76,614,550-78,186,155 | CN Gain | 1571606 | q21.13 | 0.16 | 106 |
| Em Ca | chr9:78,480,471-121,844,326 | CN Gain | 43363856 | q21.13 - q33.1 | 0.18 | 3200 |
| Em Ca | chr9:122,199,508-141,054,761 | CN Gain | 18855254 | q33.1 - q34.3 | 0.17 | 1551 |
| Em Ca | chr10:126,070-27,678,534 | CN Gain | 27552465 | p15.3 - p12.1 | 0.14 | 2059 |
| Em Ca | chr10:27,977,627-37,318,114 | CN Gain | 9340488 | p12.1 - p11.21 | 0.14 | 659 |
| Em Ca | chr10:42,930,827-51,514,824 | CN Gain | 8583998 | q11.21 - q11.23 | 0.19 | 494 |
| Em Ca | chr10:51,552,274-57,004,292 | CN Gain | 5452019 | q11.23 - q21.1 | 0.20 | 380 |
| Em Ca | chr10:57,306,282-65,908,613 | CN Gain | 8602332 | q21.1 - q21.3 | 0.21 | 618 |
| Em Ca | chr10:66,418,226-68,866,134 | CN Gain | 2447909 | q21.3 | 0.27 | 177 |
| Em Ca | chr10:69,247,842-89,376,952 | CN Gain | 20129111 | q21.3 - q23.2 | 0.19 | 1642 |
| Em Ca | chr10:89,392,727-90,311,151 | CN Loss | 918425 | q23.2 - q23.31 | -0.65 | 219 |
| Em Ca | chr10:71,376,514-135,434,303 | LOH | 64057790 | q22.1 - q26.3 | 0.18 | 4886 |
| Em Ca | chr10:90,334,835-135,434,303 | CN Gain | 45099469 | q23.31 - q26.3 | 0.19 | 3200 |
| Em Ca | chr12:189,400-20,732,241 | CN Gain | 20542842 | p13.33 - p12.2 | 0.43 | 1813 |
| Em Ca | chr12:20,751,526-21,011,235 | CN Gain | 259710 | p12.2 | 0.44 | 18 |
| Em Ca | chr12:21,027,327-89,624,279 | CN Gain | 68596953 | p12.2 - q21.33 | 0.44 | 5279 |
| Em Ca | chr12:89,640,358-90,668,725 | CN Gain | 1028368 | q21.33 | 0.36 | 69 |
| Em Ca | chr12:90,677,833-99,457,672 | CN Gain | 8779840 | q21.33 - q23.1 | 0.21 | 651 |
| Em Ca | chr12:99,741,092-110,619,797 | CN Gain | 10878706 | q23.1 - q24.11 | 0.23 | 816 |
| Em Ca | chr12:110,172,974-112,991,655 | LOH | 2818682 | q24.11 - q24.13 | 0.14 | 249 |
| Em Ca | chr12:110,996,263-112,375,308 | CN Gain | 1379046 | q24.11 - q24.13 | 0.14 | 108 |
| Em Ca | chr12:112,985,328-126,276,229 | CN Gain | 13290902 | q24.13 - q24.32 | 0.19 | 1011 |
| Em Ca | chr12:126,291,555-129,122,701 | CN Gain | 2831147 | q24.32 | 0.27 | 200 |
| Em Ca | chr12:129,140,015-133,818,115 | CN Gain | 4678101 | q24.32 - q24.33 | 0.23 | 267 |
| Em Ca | chr14:20,219,083-38,180,104 | CN Gain | 17961022 | q11.2 - q21.1 | 0.20 | 1448 |
| Em Ca | chr14:38,563,111-41,104,823 | CN Gain | 2541713 | q21.1 | 0.20 | 144 |
| Em Ca | chr14:41,108,959-41,384,295 | CN Gain | 275337 | q21.1 | 0.28 | 19 |
| Em Ca | chr14:41,408,099-62,885,208 | CN Gain | 21477110 | q21.1 - q23.2 | 0.15 | 1534 |
| Em Ca | chr14:63,191,094-67,462,202 | CN Gain | 4271109 | q23.2 - q23.3 | 0.20 | 419 |
| Em Ca | chr14:67,464,304-67,556,010 | CN Gain | 91707 | q23.3 | 0.37 | 19 |
| Em Ca | chr14:67,562,723-87,206,478 | CN Gain | 19643756 | q23.3 - q31.3 | 0.17 | 1486 |
| Em Ca | chr14:86,879,485-89,497,782 | LOH | 2618298 | q31.3 | 0.10 | 177 |
| Em Ca | chr14:89,290,630-107,282,024 | CN Gain | 17991395 | q31.3 - q32.33 | 0.18 | 1502 |
| Em Ca | chr16:28,853,996-35,271,725 | LOH | 6417730 | p11.2 - p11.1 | -0.08 | 301 |
| Em Ca | chr17:400,959-7,594,942 | CN Gain | 7193984 | p13.3 - p13.1 | 0.27 | 624 |
| Em Ca | chr17:7,596,409-7,616,383 | CN Gain | 19975 | p13.1 | 0.16 | 24 |
| Em Ca | chr17:7,617,287-35,771,468 | CN Gain | 28154182 | p13.1 - q12 | 0.30 | 1887 |
| Em Ca | chr17:35,787,666-36,043,653 | CN Gain | 255988 | q12 | 0.25 | 19 |
| Em Ca | chr17:36,045,807-48,685,792 | CN Gain | 12639986 | q12 - q21.33 | 0.29 | 1238 |
| Em Ca | chr17:48,686,782-48,703,791 | CN Gain | 17010 | q21.33 | 0.25 | 19 |
| Em Ca | chr17:48,704,511-48,739,543 | CN Gain | 35033 | q21.33 | 0.39 | 34 |
| Em Ca | chr17:48,739,634-54,957,844 | CN Gain | 6218211 | q21.33 - q22 | 0.34 | 477 |
| Em Ca | chr17:54,967,528-56,458,129 | CN Gain | 1490602 | q22 | 0.44 | 158 |
| Em Ca | chr17:56,468,569-57,133,429 | CN Gain | 664861 | q22 | 0.21 | 46 |
| Em Ca | chr17:55,703,550-59,366,049 | LOH | 3662500 | q22 - q23.2 | 0.29 | 289 |
| Em Ca | chr17:57,148,991-58,024,275 | CN Gain | 875285 | q22 - q23.1 | 0.37 | 82 |
| Em Ca | chr17:58,031,153-61,345,374 | CN Gain | 3314222 | q23.1 - q23.3 | 0.24 | 258 |
| Em Ca | chr17:61,357,532-63,798,011 | CN Gain | 2440480 | q23.3 - q24.1 | 0.30 | 218 |
| Em Ca | chr17:64,093,392-69,222,669 | CN Gain | 5129278 | q24.1 - q24.3 | 0.30 | 355 |
| Em Ca | chr17:69,236,998-69,893,058 | CN Gain | 656061 | q24.3 | 0.23 | 45 |
| Em Ca | chr17:69,904,186-80,263,427 | CN Gain | 10359242 | q24.3 - q25.3 | 0.39 | 921 |
| Em Ca | chr18:12,842-14,948,027 | CN Gain | 14935186 | p11.32 - p11.21 | 0.26 | 1055 |
| Em Ca | chr18:63,770,935-64,394,016 | CN Loss | 623082 | q22.1 | -0.19 | 42 |
| Em Ca | chr18:63,678,318-78,007,784 | LOH | 14329467 | q22.1 - q23 | -0.20 | 977 |
| Em Ca | chr18:65,220,234-78,007,784 | CN Loss | 12787551 | q22.1 - q23 | -0.20 | 878 |
| Em Ca | chr19:247,232-1,203,852 | CN Gain | 956621 | p13.3 | 0.15 | 136 |
| Em Ca | chr19:1,204,464-1,224,518 | CN Gain | 20055 | p13.3 | 0.27 | 19 |
| Em Ca | chr19:1,224,831-23,562,604 | CN Gain | 22337774 | p13.3 - p12 | 0.11 | 1899 |
| Em Ca | chr19:28,555,386-35,602,498 | CN Gain | 7047113 | q11 - q13.12 | 0.14 | 581 |
| Em Ca | chr19:35,648,365-38,514,717 | LOH | 2866353 | q13.12 - q13.13 | -0.05 | 190 |
| Em Ca | chr19:40,741,694-56,008,598 | CN Gain | 15266905 | q13.2 - q13.42 | 0.12 | 1488 |
| Em Ca | chr19:56,307,723-56,779,041 | CN Gain | 471319 | q13.43 | 0.21 | 31 |
| Em Ca | chr19:58,831,966-59,093,239 | CN Gain | 261274 | q13.43 | 0.23 | 20 |
| Em Ca | chr22:16,054,713-17,997,569 | CN Loss | 1942857 | q11.1 - q11.21 | -0.12 | 87 |
| Em Ca | chr22:21,032,419-21,266,239 | CN Loss | 233821 | q11.21 | -0.22 | 24 |
| Em Ca | chr22:22,082,919-22,257,205 | CN Loss | 174287 | q11.21 - q11.22 | -0.23 | 19 |
| Em Ca | chr22:24,346,428-24,390,318 | Homozygous Copy Loss | 43891 | q11.23 | -2.22 | 20 |
| Em Ca | chr22:28,706,220-29,100,711 | CN Loss | 394492 | q12.1 | -0.19 | 32 |
| Em Ca | chr22:33,240,290-33,511,194 | CN Loss | 270905 | q12.3 | -0.21 | 23 |
| Em Ca | chr22:41,259,463-41,661,154 | CN Loss | 401692 | q13.2 | -0.18 | 45 |
| Em Ca | chrX:7,561,105-8,018,265 | CN Gain | 457161 | p22.31 | 0.18 | 30 |
| Em Ca | chrX:48,628,844-53,764,840 | LOH | 5135997 | p11.23 - p11.22 | 0.00 | 336 |
| Em Ca | chrX:61,732,394-67,098,074 | LOH | 5365681 | q11.1 - q12 | 0.04 | 620 |
| Em Ca | chrX:65,698,132-66,674,521 | CN Gain | 976390 | q12 | 0.14 | 71 |
| Em Ca | chrX:71,303,254-75,504,972 | LOH | 4201719 | q13.1 - q13.3 | 0.05 | 307 |
| Em Ca | chrX:72,430,257-75,406,747 | CN Gain | 2976491 | q13.2 - q13.3 | 0.11 | 215 |
| Em Ca | chrX:108,695,168-115,087,323 | LOH | 6392156 | q22.3 - q23 | 0.02 | 555 |
| Em Ca | chrX:125,162,950-128,668,467 | LOH | 3505518 | q25 | -0.01 | 223 |
| Em Ca | chrX:127,005,756-127,289,763 | CN Gain | 284008 | q25 | 0.23 | 19 |
| Em Ca | chrY:2,660,163-28,799,935 | Homozygous Copy Loss | 26139773 | p11.31 - q11.23 | -3.55 | 614 |
| PMM | chr1:49,113,622-53,203,664 | LOH | 4090043 | p33 - p32.3 | -0.08 | 317 |
| PMM | chr3:42,630,225-45,177,379 | LOH | 2547155 | p22.1 - p21.31 | 0.00 | 172 |
| PMM | chr3:46,677,519-53,041,344 | LOH | 6363826 | p21.31 - p21.1 | -0.03 | 646 |
| PMM | chr3:162,481,733-162,726,432 | CN Loss | 244700 | q26.1 | -0.49 | 18 |
| PMM | chr3:162,094,173-165,917,057 | LOH | 3822885 | q26.1 | 0.01 | 255 |
| PMM | chr4:168,546,573-168,758,156 | CN Loss | 211584 | q32.3 | -0.84 | 20 |
| PMM | chr5:1,296,486-1,722,978 | CN Gain | 426493 | p15.33 | 0.52 | 59 |
| PMM | chr7:61,064,518-63,941,206 | LOH | 2876689 | q11.1 - q11.21 | -0.03 | 96 |
| PMM | chr8:49,272,709-51,888,489 | LOH | 2615781 | q11.21 | -0.01 | 176 |
| PMM | chr13:54,780,049-57,361,568 | LOH | 2581520 | q14.3 - q21.1 | -0.06 | 175 |
| PMM | chr14:86,879,485-89,497,782 | LOH | 2618298 | q31.3 | -0.06 | 177 |
| PMM | chr16:32,529,165-34,344,398 | CN Gain | 1815234 | p11.2 | 0.41 | 34 |
| PMM | chr16:31,897,308-35,271,725 | LOH | 3374418 | p11.2 - p11.1 | -0.02 | 110 |
| PMM | chr17:55,932,988-59,366,049 | LOH | 3433062 | q22 - q23.2 | -0.01 | 266 |
| PMM | chr22:24,347,758-24,394,088 | Homozygous Copy Loss | 46331 | q11.23 | -1.89 | 19 |
| PMM | chrX:48,628,844-54,296,244 | LOH | 5667401 | p11.23 - p11.22 | -0.04 | 371 |
| PMM | chrX:61,732,394-67,085,251 | LOH | 5352858 | q11.1 - q12 | -0.03 | 619 |
| PMM | chrX:71,870,482-75,504,972 | LOH | 3634491 | q13.2 - q13.3 | 0.01 | 248 |
| PMM | chrX:96,002,038-98,912,783 | LOH | 2910746 | q21.33 - q22.1 | -0.07 | 191 |
| PMM | chrX:109,452,851-114,742,466 | LOH | 5289616 | q23 | -0.03 | 466 |
| PMM | chrX:125,751,389-128,289,349 | LOH | 2537961 | q25 | -0.05 | 159 |
| PMM | chrY:2,660,163-28,799,935 | Homozygous Copy Loss | 26139773 | p11.31 - q11.23 | -3.72 | 614 |
