# Supplementary material for: Molecular characteristics of endometrial cancer coexisting with peritoneal malignant mesothelioma in Li-Fraumeni-like syndrome
Source: BMC Cancer. 2015 Jan 15;15:8. doi: 10.1186/s12885-015-1010-x (PMC4312462; doi:10.1186/s12885-015-1010-x)
Supplement: Additional file 3: — Interpretation of somatic mutation with Affymetrix OncoScan microarrays. [file 12885_2015_1010_MOESM3_ESM.doc]

**Additional file 3. Interpretation of somatic mutation with Affymetrix OncoScan microarrays.**

The OncoScan microarray contains probes to analyze 76 somatic mutations that commonly occur in cancer, as listed in the following table. The procedures of identification of somatic mutation are as follows. First, CEL file of a microarray is converted to OSCHP file using OncoScan Console, which is freely provided by Affymetrix. Second, both tiers of criteria should be fulfilled to determine the mutation call (Mut Call):

(1) Using Somatic Mutation Viewer (version 1.01) that is freely provided by Affymetrix, data of 76 mutations were coded with grey color (undetected), blue color (low confidence), and red color (high confidence), as shown in the following Figure. Of note, the farthest right mark was the very high signal intensity of PIK3CA mutation 1623G>A.


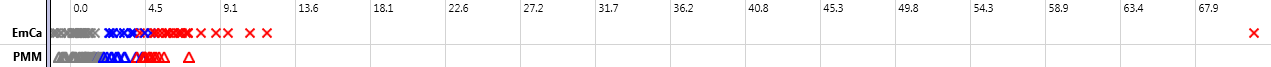


(2) The OncoScan console provided the value of ndSNPQC, which is a measure of how well genotype alleles are resolved in the microarray data. Based on the value of ndSNPQC, three different actions should be taken, as described as follows.

| **ndSNPQC <26**  **(shown as marker: x)** | **26 < ndSNPQC < 34**  **(shown as the marker: △)** | **ndSNPQC > 34**  **(shown as the marker: +)** |
| --- | --- | --- |
| Somatic mutations called for the samples in this range should be independently validated. | Calls made for the samples in this range can be reliable if significantly separated, but they need to be manually examined. | In these samples of good quality, all high confidence calls should be trusted. |

**Table: List of 76 common somatic mutations identified with OncoScan microarray and the results of the endometrial cancer tissues in this case (*).**

| **Chromosome**   |  | **Event** | **Type** | **Common Name** | **mut_nucleotide** | **mut_amino acid** | **Mut Call*** | | --- | --- | --- | --- | --- | --- | --- | | chr1:115,256,529-115,256,529 | Point Mutation | Missense | NRAS:p.Q61L:c.182A>T | a>t | Q>L | undetected | | chr1:115,256,529-115,256,529 | Point Mutation | Missense | NRAS:p.Q61R:c.182A>G | a>g | Q>R | undetected | | chr1:115,256,530-115,256,530 | Point Mutation | Missense | NRAS:p.Q61K:c.181C>A | c>a | Q>K | undetected | | chr1:115,258,747-115,258,747 | Point Mutation | Missense | NRAS:p.G12D:c.35G>A | g>a | G>D | undetected | | chr1:115,258,747-115,258,747 | Point Mutation | Missense | NRAS:p.G12V:c.35G>T | g>t | G>V | undetected | | chr1:115,258,748-115,258,748 | Point Mutation | Missense | NRAS:p.G12S/C:c.34G>A/T | g>a | G>S | undetected | | chr1:115,258,748-115,258,748 | Point Mutation | Missense | NRAS:p.G12S/C:c.34G>A/T | g>t | G>C | undetected | | chr2:209,113,112-209,113,112 | Point Mutation | Missense | IDH1:p.R132H:c.395G>A | g>a | R>H | undetected | | **chr3:178,936,082-178,936,082** | **Point Mutation** | **Missense** | **PIK3CA:p.E542K:c.1624G>A** | **g>a** | **E>K** | **High confidence** | | chr3:178,936,091-178,936,091 | Point Mutation | Missense | PIK3CA:p.E545K:c.1633G>A | g>a | E>K | undetected | | chr3:178,936,094-178,936,094 | Point Mutation | Missense | PIK3CA:p.Q546K:c.1636C>A | c>a | Q>K | undetected | | chr3:178,952,085-178,952,085 | Point Mutation | Missense | PIK3CA:p.H1047R:c.3140A>G | a>g | H>R | undetected | | chr3:178,952,085-178,952,085 | Point Mutation | Missense | PIK3CA:p.H1047L:c.3140A>T | a>t | H>L | undetected | | chr7:55,241,707-55,241,707 | Point Mutation | Missense | EGFR:p.G719S:c.2155G>A | g>a | G>S | undetected | | chr7:55,241,707-55,241,707 | Point Mutation | Missense | EGFR:p.G719C:c.2155G>T | g>t | G>C | undetected | | chr7:55,241,708-55,241,708 | Point Mutation | Missense | EGFR:p.G719A:c.2156G>C | g>c | G>A | undetected | | chr7:55,242,465-55,242,479 | Deletion | In-Frame | EGFR:p.E746_A750del:c.2235_2249del15 | ggaattaagagaagc>- | ELREA>- | undetected | | chr7:55,242,466-55,242,480 | Deletion | In-Frame | EGFR:p.E746_A750del:c.2236_2250del15 | gaattaagagaagca>- | ELREA>- | undetected | | chr7:55,242,469-55,242,477 | Deletion | In-Frame | EGFR:p.L747_E749P/del:c.2239_2248>C/G | ttaagagaa>- | LRE>- | undetected | | chr7:55,242,467-55,242,481 | Seq. Variant | Deletion In-Frame | EGFR:p.E746_T751>A:c.2237_2251del15 | aattaagagaagcaa>- | ELREAT>A | undetected | | chr7:55,242,469-55,242,478 | Seq. Variant | Deletion In-Frame | EGFR:p.L747_E749P/del:c.2239_2248>C/G | ttaagagaag>c | LREA>P | undetected | | chr7:55,242,468-55,242,482 | Deletion | In-Frame | EGFR:p.L747_T751del:c.2240_2254del15 | attaagagaagcaac>- | LREAT>- | undetected | | chr7:55,242,469-55,242,483 | Deletion | In-Frame | EGFR:p.L747_T751del:c.2240_2254del15 | ttaagagaagcaaca>- | LREAT>- | undetected | | chr7:55,242,470-55,242,484 | Deletion | In-Frame | EGFR:p.L747_T751del:c.2240_2254del15 | taagagaagcaacat>- | LREAT>- | undetected | | chr7:55,242,470-55,242,487 | Seq. Variant | Deletion In-Frame | EGFR:p.L747_P753>S:c.2240_2257del18 | taagagaagcaacatctc>- | LREATSP>S | undetected | | chr7:55,249,009-55,249,010 | Insertion | In-Frame | EGFR:p.V769_D770insASV:c.2307_2308ins9 | ->gccagcgtg | ->ASV | undetected | | chr7:55,249,013-55,249,014 | Insertion | In-Frame | EGFR:p.D770_N771insSVD:c.2311_2312ins9 | ->gcgtggaca | ->SVD | undetected | | chr7:55,249,021-55,249,022 | Insertion | In-Frame | EGFR:p.H773_V774insNPH:c.2319_2320ins9 | ->aacccccac | ->NPH | undetected | | chr7:55,249,071-55,249,071 | Point Mutation | Missense | EGFR:p.T790M:c.2369C>T | c>t | T>M | undetected | | chr7:55,259,515-55,259,515 | Point Mutation | Missense | EGFR:p.L858R:c.2573T>G | t>g | L>R | undetected | | chr7:55,259,524-55,259,524 | Point Mutation | Missense | EGFR:p.L861Q:c.2582T>A | t>a | L>Q | undetected | | chr7:140,453,136-140,453,136 | Point Mutation | Missense | BRAF:p.V600E:c.1799T>A | t>a | V>E | undetected | | chr7:140,453,136-140,453,137 | Point Mutation | Missense | BRAF:p.V600K:c.1798_1799GT>AA | gt>aa | V>K | undetected | | chr7:140,481,402-140,481,402 | Point Mutation | Missense | BRAF:p.G469A:c.1406G>C | g>c | G>A | undetected | | chr7:140,481,402-140,481,402 | Point Mutation | Missense | BRAF:p.G469E:c.1406G>A | g>a | G>E | undetected | | chr10:89,692,904-89,692,904 | Point Mutation | Nonsense | PTEN:p.R130*:c.388C>T | c>t | R>* | undetected | | chr10:89,692,904-89,692,904 | Point Mutation | Missense | PTEN:p.R130G:c.388C>G | c>g | R>G | undetected | | chr10:89,692,905-89,692,905 | Point Mutation | Missense | PTEN:p.R130Q/fs*4:c.389G>A/delG | g>a | R>Q | undetected | | chr10:89,692,905-89,692,905 | Deletion | Frame-Shift | PTEN:p.R130Q/fs*4:c.389G>A/delG | g>- | ->QLV* | undetected | | chr10:89,692,993-89,692,993 | Point Mutation | Missense | PTEN:p.R159S:c.477G>T | g>t | R>S | undetected | | chr10:89,717,672-89,717,672 | Point Mutation | Nonsense | PTEN:p.R233*:c.697C>T | c>t | R>* | undetected | | chr10:89,717,716-89,717,717 | Insertion | Frame-Shift | PTEN:p.P248fs*5:c.741_742insA | ->a | ->TCVW* | undetected | | chr10:89,717,775-89,717,775 | Deletion | Frame-Shift | PTEN:p.K267fs*9:c.800delA | a>- | ->RTKCFTFG* | undetected | | chr12:25,378,562-25,378,562 | Point Mutation | Missense | KRAS:p.A146P:c.436G>C | g>c | A>P | undetected | | chr12:25,380,275-25,380,275 | Point Mutation | Missense | KRAS:p.Q61H:c.183A>T | a>t | Q>H | undetected | | chr12:25,380,275-25,380,275 | Point Mutation | Missense | KRAS:p.Q61H:c.183A>C | a>c | Q>H | undetected | | chr12:25,380,277-25,380,277 | Point Mutation | Missense | KRAS:p.Q61K/K:c.180_181TC>TA/AA | c>a | Q>K | undetected | | chr12:25,380,277-25,380,278 | Point Mutation | Missense | KRAS:p.Q61K/K:c.180_181TC>TA/AA | tc>aa | Q>K | undetected | | chr12:25,398,281-25,398,281 | Point Mutation | Missense | KRAS:p.G13D:c.38G>A | g>a | G>D | undetected | | chr12:25,398,284-25,398,284 | Point Mutation | Missense | KRAS:p.G12D/V:c.35G>A/T | g>a | G>D | undetected | | chr12:25,398,284-25,398,284 | Point Mutation | Missense | KRAS:p.G12D/V:c.35G>A/T | g>t | G>V | undetected | | chr12:25,398,284-25,398,284 | Point Mutation | Missense | KRAS:p.G12A:c.35G>C | g>c | G>A | undetected | | chr12:25,398,285-25,398,285 | Point Mutation | Missense | KRAS:p.G12C/S:c.34G>T/A | g>t | G>C | undetected | | chr12:25,398,285-25,398,285 | Point Mutation | Missense | KRAS:p.G12C/S:c.34G>T/A | g>a | G>S | undetected | | chr15:90,631,838-90,631,838 | Point Mutation | Missense | IDH2:p.R172K:c.515G>A | g>a | R>K | undetected | | chr15:90,631,934-90,631,934 | Point Mutation | Missense | IDH2:p.R140Q:c.419G>A | g>a | R>Q | undetected | | chr17:7,577,022-7,577,022 | Point Mutation | Nonsense | TP53:p.R306*:c.916C>T | c>t | R>* | undetected | | chr17:7,577,094-7,577,094 | Point Mutation | Missense | TP53:p.R282W:c.844C>T | c>t | R>W | undetected | | chr17:7,577,120-7,577,120 | Point Mutation | Missense | TP53:p.R273H/L:c.818G>A/T | g>t | R>L | undetected | | chr17:7,577,120-7,577,120 | Point Mutation | Missense | TP53:p.R273H/L:c.818G>A/T | g>a | R>H | undetected | | chr17:7,577,121-7,577,121 | Point Mutation | Missense | TP53:p.R273C/S:c.817C>T/A | c>a | R>S | undetected | | chr17:7,577,121-7,577,121 | Point Mutation | Missense | TP53:p.R273C/S:c.817C>T/A | c>t | R>C | undetected | | chr17:7,577,534-7,577,534 | Point Mutation | Missense | TP53:p.R249S:c.747G>T | g>t | R>S | undetected | | chr17:7,577,538-7,577,538 | Point Mutation | Missense | TP53:p.R248Q/L:c.743G>A/T | g>t | R>L | undetected | | chr17:7,577,538-7,577,538 | Point Mutation | Missense | TP53:p.R248Q/L:c.743G>A/T | g>a | R>Q | undetected | | chr17:7,577,539-7,577,539 | Point Mutation | Missense | TP53:p.R248W:c.742C>T | c>t | R>W | undetected | | chr17:7,577,548-7,577,548 | Point Mutation | Missense | TP53:p.G245S/C:c.733G>A/T | g>a | G>S | undetected | | chr17:7,577,548-7,577,548 | Point Mutation | Missense | TP53:p.G245S/C:c.733G>A/T | g>t | G>C | undetected | | chr17:7,578,190-7,578,190 | Point Mutation | Missense | TP53:p.Y220C:c.659A>G | a>g | Y>C | undetected | | chr17:7,578,212-7,578,212 | Point Mutation | Nonsense | TP53:p.R213*:c.637C>T | c>t | R>* | undetected | | chr17:7,578,263-7,578,263 | Point Mutation | Nonsense | TP53:p.R196*:c.586C>T | c>t | R>* | undetected | | chr17:7,578,394-7,578,394 | Point Mutation | Missense | TP53:p.H179R:c.536A>G | a>g | H>R | undetected | | chr17:7,578,403-7,578,403 | Point Mutation | Missense | TP53:p.C176F:c.527G>T | g>t | C>F | undetected | | chr17:7,578,406-7,578,406 | Point Mutation | Missense | TP53:p.R175H:c.524G>A | g>a | R>H | undetected | | chr17:7,578,442-7,578,442 | Point Mutation | Missense | TP53:p.Y163C:c.488A>G | a>g | Y>C | undetected | | chr17:7,578,461-7,578,461 | Point Mutation | Missense | TP53:p.V157F:c.469G>T | g>t | V>F | undetected | |  |  |  |  |  |  |
| --- | --- | --- | --- | --- | --- | --- | --- | --- | --- | --- | --- | --- | --- | --- | --- | --- | --- | --- | --- | --- | --- | --- | --- | --- | --- | --- | --- | --- | --- | --- | --- | --- | --- | --- | --- | --- | --- | --- | --- | --- | --- | --- | --- | --- | --- | --- | --- | --- | --- | --- | --- | --- | --- | --- | --- | --- | --- | --- | --- | --- | --- | --- | --- | --- | --- | --- | --- | --- | --- | --- | --- | --- | --- | --- | --- | --- | --- | --- | --- | --- | --- | --- | --- | --- | --- | --- | --- | --- | --- | --- | --- | --- | --- | --- | --- | --- | --- | --- | --- | --- | --- | --- | --- | --- | --- | --- | --- | --- | --- | --- | --- | --- | --- | --- | --- | --- | --- | --- | --- | --- | --- | --- | --- | --- | --- | --- | --- | --- | --- | --- | --- | --- | --- | --- | --- | --- | --- | --- | --- | --- | --- | --- | --- | --- | --- | --- | --- | --- | --- | --- | --- | --- | --- | --- | --- | --- | --- | --- | --- | --- | --- | --- | --- | --- | --- | --- | --- | --- | --- | --- | --- | --- | --- | --- | --- | --- | --- | --- | --- | --- | --- | --- | --- | --- | --- | --- | --- | --- | --- | --- | --- | --- | --- | --- | --- | --- | --- | --- | --- | --- | --- | --- | --- | --- | --- | --- | --- | --- | --- | --- | --- | --- | --- | --- | --- | --- | --- | --- | --- | --- | --- | --- | --- | --- | --- | --- | --- | --- | --- | --- | --- | --- | --- | --- | --- | --- | --- | --- | --- | --- | --- | --- | --- | --- | --- | --- | --- | --- | --- | --- | --- | --- | --- | --- | --- | --- | --- | --- | --- | --- | --- | --- | --- | --- | --- | --- | --- | --- | --- | --- | --- | --- | --- | --- | --- | --- | --- | --- | --- | --- | --- | --- | --- | --- | --- | --- | --- | --- | --- | --- | --- | --- | --- | --- | --- | --- | --- | --- | --- | --- | --- | --- | --- | --- | --- | --- | --- | --- | --- | --- | --- | --- | --- | --- | --- | --- | --- | --- | --- | --- | --- | --- | --- | --- | --- | --- | --- | --- | --- | --- | --- | --- | --- | --- | --- | --- | --- | --- | --- | --- | --- | --- | --- | --- | --- | --- | --- | --- | --- | --- | --- | --- | --- | --- | --- | --- | --- | --- | --- | --- | --- | --- | --- | --- | --- | --- | --- | --- | --- | --- | --- | --- | --- | --- | --- | --- | --- | --- | --- | --- | --- | --- | --- | --- | --- | --- | --- | --- | --- | --- | --- | --- | --- | --- | --- | --- | --- | --- | --- | --- | --- | --- | --- | --- | --- | --- | --- | --- | --- | --- | --- | --- | --- | --- | --- | --- | --- | --- | --- | --- | --- | --- | --- | --- | --- | --- | --- | --- | --- | --- | --- | --- | --- | --- | --- | --- | --- | --- | --- | --- | --- | --- | --- | --- | --- | --- | --- | --- | --- | --- | --- | --- | --- | --- | --- | --- | --- | --- | --- | --- | --- | --- | --- | --- | --- | --- | --- | --- | --- | --- | --- | --- | --- | --- | --- | --- | --- | --- | --- | --- | --- | --- | --- | --- | --- | --- | --- | --- | --- | --- | --- | --- | --- | --- | --- | --- | --- | --- | --- | --- | --- | --- | --- | --- | --- | --- | --- | --- | --- | --- | --- | --- | --- | --- | --- | --- | --- | --- | --- | --- | --- | --- | --- | --- | --- | --- | --- | --- | --- | --- | --- | --- | --- | --- | --- | --- | --- | --- | --- | --- | --- | --- | --- | --- | --- |
|  |  |  |  |  |  |  |
